# Multiscale photoacoustic tomography of neural activities with GCaMP calcium indicators

**DOI:** 10.1117/1.JBO.27.9.096004

**Published:** 2022-09-10

**Authors:** Ruiying Zhang, Lei S. Li, Bin Rao, Haoyang Rong, Min-Yu Sun, Junjie Yao, Ruimin Chen, Qifa Zhou, Steven Mennerick, Baranidharan Raman, Lihong V. Wang

**Affiliations:** a Washington University in Saint Louis, Department of Biomedical Engineering, Saint Louis, Missouri, United States; b California Institute of Technology, Caltech Optical Imaging Laboratory, Andrew and Peggy Cherng Department of Medical Engineering, Department of Electrical Engineering, Pasadena, California, United States; c Washington University School of Medicine, Department of Psychiatry, Saint Louis, Missouri, United States; d University of Southern California, Department of Biomedical Engineering, Los Angeles, California, United States

**Keywords:** photoacoustic tomography, neural imaging, calcium indicators, GCaMP

## Abstract

**Significance:**

Optical imaging of responses in fluorescently labeled neurons has progressed significantly in recent years. However, there is still a need to monitor neural activities at divergent spatial scales and at depths beyond the optical diffusion limit.

**Aim:**

To meet these needs, we aim to develop multiscale photoacoustic tomography (PAT) to image neural activities across spatial scales with a genetically encoded calcium indicator GCaMP.

**Approach:**

First, using photoacoustic microscopy, we show that depth-resolved GCaMP signals can be monitored *in vivo* from a fly brain in response to odor stimulation without depth scanning and even with the cuticle intact. *In vivo* monitoring of GCaMP signals was also demonstrated in mouse brains. Next, using photoacoustic computed tomography, we imaged neural responses of a mouse brain slice at depths beyond the optical diffusion limit.

**Results:**

We provide the first unambiguous demonstration that multiscale PAT can be used to record neural activities in transgenic flies and mice with select neurons expressing GCaMP.

**Conclusions:**

Our results indicate that the combination of multiscale PAT and fluorescent neural activity indicators provides a methodology for imaging targeted neurons at various scales.

## 1 Introduction

Monitoring the neural activity of large populations of neurons with fine spatial and temporal resolution is arguably one of the most important challenges in neuroscience.^1^^–^^3^ To achieve this goal, several multiunit electrophysiological recording techniques^4^^–^^6^ and optical imaging methodologies^7^^–^^9^ have been developed in recent decades. Electrophysiological recording methods are still considered the gold standard because they directly monitor intracellular or extracellular neural signals from individual neurons or small groups of neurons with high sensitivity and temporal fidelity.^10^ Complementing these techniques, noninvasive optical imaging techniques that allow for larger spatial coverage with finer spatial resolution have been used to study computations performed by neural networks.^7^^,^^11^^,^^12^ Combined with fluorescent calcium and voltage indicators, multiphoton microscopy, including wide-field two-photon microscopy,^13^^–^^15^ Bessel-beam two-photon microscopy^16^^–^^18^ and three-photon microscopy,^19^^–^^22^ has enabled optical mapping of neural circuits from hundreds to thousands of neurons, with high spatiotemporal resolution in optically scattering brain tissue.^23^^,^^24^ However, several limitations still exist. For example, the point-by-point scanning of confocal microscopy and two-photon microscopy creates a trade-off between spatial coverage and temporal resolution. More importantly, light scattering in the neural tissue fundamentally restricts the current optical neural imaging methods to the superficial tissue layer (∼1  mm).^25^^,^^26^ To address this issue, invasive techniques using deeply implanted fiber-optic probes have been recently used to image nonsuperficial regions inside mouse brains.^27^^–^^30^ Therefore, there is a strong need for noninvasive optical imaging methods that can break the optical diffusion limit and provide rapid depth-resolved monitoring of neural activity.

Alternative to conventional optical imaging methods, photoacoustic tomography (PAT) has been demonstrated as a powerful brain imaging technique capable of providing high-resolution images with optical absorption contrast at depths far beyond the optical diffusion limit.^31^^–^^34^ Briefly, PAT is based on the photoacoustic (PA) effect, which starts with optical absorption by molecules in tissue and ends with ultrasonic emission through thermoelastic expansion.^35^ Similar to ultrasound imaging, PAT has inherent depth-resolved imaging capability based on the acoustic arrival time or acoustic focusing. Because PAT is sensitive to optical absorption contrast (both endogenous and exogenous), it is well suited for functional, molecular, and metabolic brain imaging.^36^^–^^40^ In addition, PAT is highly scalable in its spatial resolution, penetration depth and imaging speed. Previous endeavors in PA brain studies have focused on measuring hemodynamic parameters, including the blood vessel diameter, blood flow, total hemoglobin concentration, and oxygenation saturation of hemoglobin,^41^^–^^50^ which are usually slow and indirect indicators of neural activities through neurovascular coupling.^51^ In contrast, intracellular calcium signals respond to neural activities faster than hemodynamic signals and can be recorded optically with the help of intracellular calcium indicators.^52^ Among all of the calcium indicators, GCaMP, with its relatively fast conformational change upon calcium binding during action potentials, is by far the most widely used genetically encoded calcium-sensitive protein.^53^^,^^54^ The conformational change of GCaMP results in a substantial increase in its optical absorption, which is ideal for PAT imaging of calcium signals with high sensitivity. However, it is yet to be determined whether experimental results support this hypothesis and how the PA signals compare to the fluorescence signals.

Here, for the first time, we clearly demonstrate that PAT can be used to monitor neural activities in select neurons expressing GCaMP indicators in transgenic invertebrate (fly) and vertebrate (mouse) models. To accommodate different spatial scales, we adapted two PAT embodiments—photoacoustic microscopy (PAM) and photoacoustic computed tomography (PACT). With PAM, we demonstrate high-resolution depth-resolved neural imaging without depth scanning, which is desired for monitoring neural activities with high temporal resolution. With PACT, we demonstrate wide-field imaging of GCaMP calcium signals at depths beyond the optical diffusion limit. The combination of PAT and GCaMP or other potential calcium indicators provides a promising platform for functional brain imaging at different spatial scales.

## 2 Materials and Methods

### 2.1 PAT of Neuronal Calcium Signals with GCaMP

In PAT, as tissues are excited by laser pulses, laser energy is absorbed by biomolecules, and converted through both the fluorescence channel by radiative relaxation and the PA channel by nonradiative relaxation. An ultrasonic transducer or transducer array is used to detect the PA waves as they propagate out of the tissue. The detected PA signals are used to reconstruct an image that maps the original optical energy deposition due to nonradiative relaxation in the tissue.

Based on the image formation methods, PAT has two major implementations. The first combines mechanical scanning of a focused single-element ultrasonic transducer with direct image formation and is commonly used in PAM. The second combines parallel detection by a multielement ultrasonic transducer array or a mechanical/electronic scanning equivalent with image reconstruction and is used in PACT. In both PAM and PACT, the PA amplitude is proportional to the optical absorption coefficient of the absorber in the unit of ${\mathrm{cm}}^{-1}$, its nonradiative quantum yield (approximately equal to 1 – the fluorescence quantum yield if the photochemical channel is negligible), and the local optical fluence (or exposure) in the unit of $J/m^{2}$.

Two GCaMP variants were used in this study: GCaMP5G for fly brain imaging and GCaMP6f for mouse brain slice imaging. In general, GCaMP consists of a circularly permuted EGFP, an M13 fragment of myosin light chain kinase, and a calcium binding site of calmodulin. Action potentials trigger a large and rapid influx of ${\mathrm{Ca}}^{2+}$.^55^ In the presence of calcium, calmodulin interacts with the M13 fragment, eliciting a conformational change in EGFP and resulting in an increase in its optical absorption coefficient.^56^^,^^57^ Because the PA signal of GCaMP is proportional to its absorption coefficient, PAT has a 100% sensitivity to the absorption change of GCaMP upon the binding of ${\mathrm{Ca}}^{2+}$.

### 2.2 PAM of the Fly Brain *in Vivo*

**Fig. 1:**
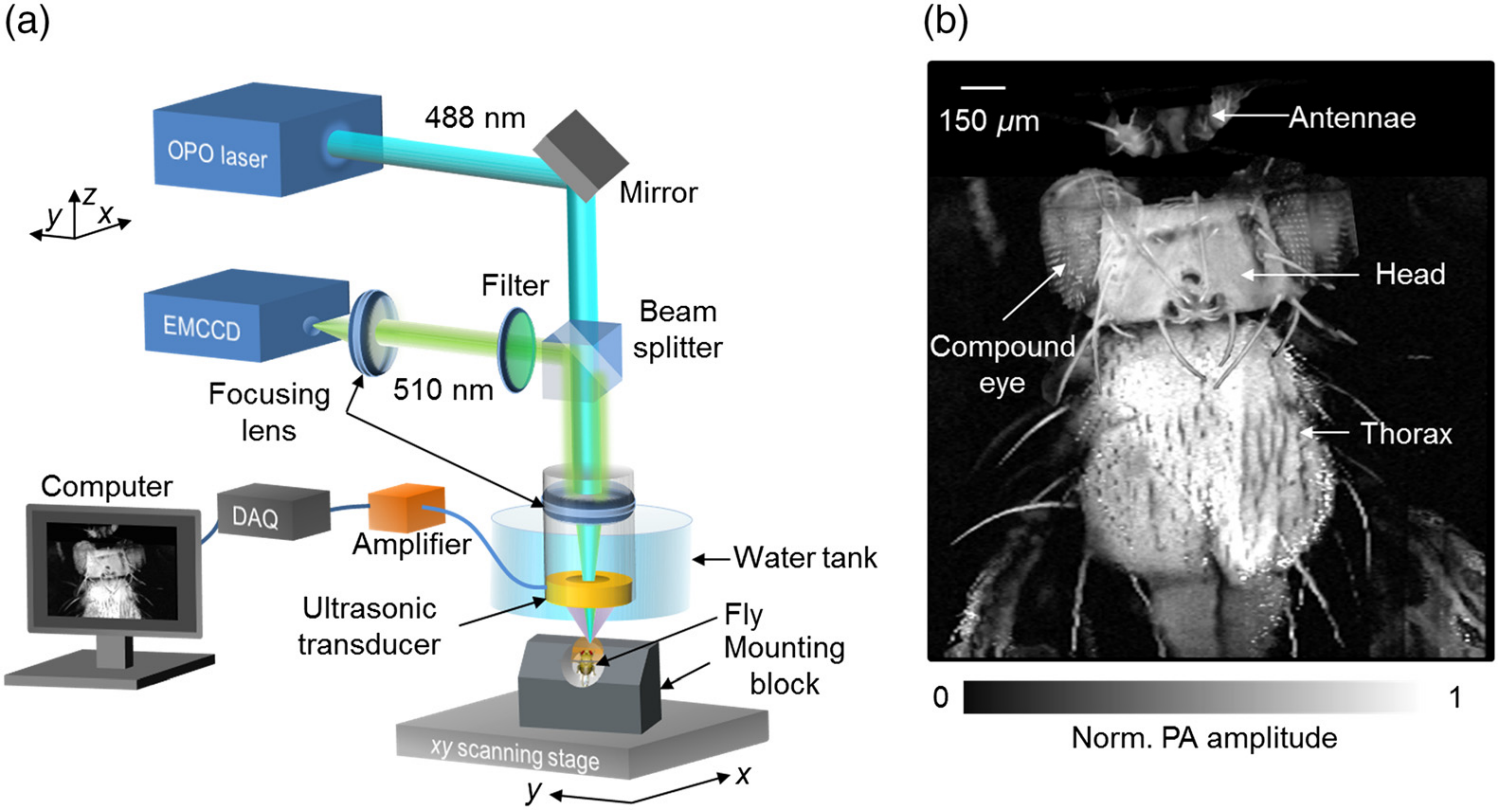
PAM of the GCaMP5G-expressing fly brain. (a) Schematic of the dual-modality PAM and epifluorescence microscopy (EFM) system. PAM and EFM share the same light path for excitation. The photoacoustic (PA) signal is detected by a ring-shaped ultrasonic transducer, whereas the fluorescence signal is detected by an EMCCD. (b) PAM image of a fly with endogenous contrast, showing the major parts of the fly, including the antennae, compound eyes, head, and thorax. The PA signals mostly come from pigments (e.g., melanin) on the fly cuticle.

A dual-modality PA and epifluorescence microscopy system shown schematically in Fig. 1(a) was used to image the fly brain. A tunable OPO laser (a 1-kHz pulse repetition rate, 5-ns pulse width; NT242-SH, Ekspla, Ltd.) provided 488-nm wavelength excitation light, which was collimated and coupled into a miniature imaging probe containing an achromatic focusing lens with a 13-mm focal length and a customized ring-shaped ultrasonic transducer (a 40-MHz central frequency, ∼100% bandwidth, and 6.2-mm focal length). The laser beam formed a 6-μm optical focal spot confocally aligned with the acoustic focus of the ring transducer. The axial resolution of the PAM, determined by the ultrasonic transducer bandwidth, was calculated to be ∼21  μm.^58^ A beam splitter reflected the fluorescent light for fluorescence imaging. The fluorescent light passed through another focusing lens (a 50-mm focal length) and a bandpass filter (a 525-nm central wavelength, 39-nm bandwidth; Thorlabs, Inc.) before being collected by an EMCCD camera (iXon Ultra 888, Andor, Ltd.). PA signals were sampled by a 200-MHz data acquisition (DAQ) system (National Instruments, Corp.), and fluorescence signals were recorded by the EMCCD camera’s onboard DAQ system. For each M-mode recording, PA signals before and after odor stimulation from a volume (∼6  μm×∼6  μm×∼200  μm) of neurons inside the brain tissue were acquired at a one-dimensional (1D) imaging rate of 1 kHz. To form a three-dimensional (3D) image of the fly brain, two-dimensional (2D) raster scanning was performed by motor stages. A photodiode (not shown) was used to compensate for the laser pulse energy fluctuation.

### 2.3 Fly Preparation and Odor Stimulation

GCaMP5G flies were purchased from the Bloomington Drosophila Stock Center at Indiana University. Flies were anesthetized by ${\mathrm{CO}}_{2}$ until their movement stopped and were moved to a customized fly mount (a block setup^59^ or a foil setup^60^). For the block setup, the fly was mounted and head-fixed using two-component silicone glue (KWIK-SIL, World Precision Instruments, Inc.). A thin wire was used to pull the antennae plate forward and make space between the antennae plate and the head. A small rectangular hole was created on the bottom of a 60  mm×15  mm petri dish (Falcon^®^, Corning, Inc.). Four sections of double-sided tape were used to create a rectangular window slightly larger than the head of the fly. The petri dish with the small rectangular open window at the bottom was placed on top of the fly’s head and fixed onto the mounting block by double-sided tape. The interior rectangular edges were then sealed with two-component silicone glue. For the foil setup, a piece of aluminum foil was glued to the top of a supporting plate. A 60  mm×15  mm Petri dish (Falcon^®^, Corning, Inc.) with its bottom removed was attached to the top of the supporting plate to form a saline chamber. A hole was made in the foil according to the fly’s size. The fly was then wedged into the hole with its dorsal side facing up. The gap between the fly and foil was sealed using epoxy (Permatex, Inc.). The ventral side, especially the antennae, of the fly was kept underneath the foil. The fly was then transferred to the PA/fluorescence imaging setup for experiments with the cuticle intact.

For experiments with the cuticle removed, a fine forceps (Dumont #5sf, Fine Science Tools, Inc.) was used to cut and remove the cuticle above the brain. Connective tissue and the trachea above the brain were also removed. The petri dish was then filled with fly Ringer’s solution (103 mM NaCl, 3 mM KCl, 5 mM TES, 10 mM trehalose, 10 mM glucose, 9 mM sucrose, 26 mM ${\mathrm{NaHCO}}_{3}$, 1 mM ${\mathrm{NaH}}_{2}{\mathrm{PO}}_{4}$, 1.5 mM ${\mathrm{CaCl}}_{2}$, and 4 mM ${\mathrm{MgCl}}_{2}$, adjusted to 275–280 mOsm, bubbled with 95% $O_{2}/5\%{\mathrm{CO}}_{2}$) for acoustic coupling. Note that both during the preparation and during olfactory stimulation experiments, the antennae remained completely dry. A constant airflow (carrier stream; 0.8  L/min) was maintained across the fly antenna. For odor stimulation, a constant volume of headspace (0.5  L/min) above an odor bottle containing ethyl acetate was injected into the carrier stream.

### 2.4 PACT of Acute Mouse Brain Slices

An optical parametric oscillator (OPO) laser (basiScan, Spectra-Physics, Inc.), pumped by an Nd: YAG laser (Qsmart 850, Quantel, Inc.), provided 488-nm-wavelength pulses with a 10-Hz pulse repetition rate. The laser beam was homogenized by an optical diffuser (EDC-5, RPC Photonics, Inc.). The PA signals were detected by a full-ring ultrasonic transducer array (Imasonic, Inc.) with a 10-cm diameter, 5-MHz central frequency, >90% one-way bandwidth, and 512 elements. Each element (20-mm height, 0.61-mm pitch, and 0.1-mm interelement space) was cylindrically focused to produce an axial focal length of 40 mm (acoustic NA: 0.25). The combined foci of all 512 elements formed a field of view of ∼20  mm diameter with an approximately isotropic in-plane resolution of ∼100  μm and a elevational resolution of ∼1  mm. The wide-field imaging exposure time was under 20  μs with a frame rate of 10 Hz. The raw data from each element was first Wiener deconvolved to account for the ultrasonic transducer’s impulse response and then reconstructed based on the dual speed-of-sound half-time backprojection algorithm to minimize acoustic heterogeneity.^61^^–^^63^

A brain slice holder made of 4% agarose was placed in the center of the imaging chamber of the PACT system. A silicone tube was used for high-potassium perfusion (150 mM KCl concentration) in the imaging chamber. During imaging, the chamber was filled with a regular artificial cerebrospinal fluid (aCSF) solution and continuously bubbled with 5% ${\mathrm{CO}}_{2}$ and 95% $O_{2}$. A peristaltic pump circulated and homogenized the aCSF solution within the imaging chamber.

### 2.5 Brain Slice Preparation

The protocol for mouse brain slice preparation was approved by the Animal Studies Committee at Washington University in St. Louis. Acute coronal brain slices were prepared from 3 months old GCaMP6f (C57BL/6J-Tg) mice, which were purchased from Jackson Laboratory. The GCaMP6f labeled mice were anesthetized with isoflurane and decapitated. The brain was removed and glued onto a vibratome (Leica VT1200s, Leica Microsystems, Inc.) specimen holder. Coronal slices (300-μm thick) were cut in an ice-cold modified aCSF solution (125-mM NaCl, 25-mM glucose, 25-mM ${\mathrm{NaHCO}}_{3}$, 2.5-mM KCl, 1.25-mM ${\mathrm{NaH}}_{2}{\mathrm{PO}}_{4}$, 0.5-mM ${\mathrm{CaCl}}_{2}$, and 3-mM ${\mathrm{MgCl}}_{2}$) equilibrated with 5% ${\mathrm{CO}}_{2}$ and 95% $O_{2}$. Slices were then transferred to a choline-based aCSF solution (92-mM choline chloride, 2.5-mM KCl, 1.2-mM NaH2PO4, 30-mM NaHCO3, 20-mM HEPES, 25-mM glucose, 5-mM Na-ascorbate, 2-mM thiourea, 3-mM Na-pyruvate, 2-mM ${\mathrm{CaCl}}_{2}$, and 1-mM ${\mathrm{MgCl}}_{2}$) equilibrated with 5% ${\mathrm{CO}}_{2}$ and 95% $O_{2}$, incubated at 32°C for 30 min, subsequently stored in a regular aCSF solution (125-mM NaCl, 25-mM glucose, 25-mM ${\mathrm{NaHCO}}_{3}$, 2.5-mM KCl, 1.25-mM ${\mathrm{NaH}}_{2}{\mathrm{PO}}_{4}$, 2-mM ${\mathrm{CaCl}}_{2}$, and 1-mM ${\mathrm{MgCl}}_{2}$) equilibrated with 5% ${\mathrm{CO}}_{2}$ and 95% $O_{2}$ at room temperature for more than an hour, and imaged with the PACT system.

### 2.6 Wide-field Fluorescence Imaging of the Fly Brain and Acute Mouse Brain Slices

Wide-field fluorescence imaging was performed on an Olympus Fluoview 1000 system (excitation wavelength at 488 nm, emission filter wavelength centered at 510 nm; Olympus, Corp.). The fluorescence images were captured by an EMCCD camera (iXon Ultra 888, Andor, Ltd.). A 10× water immersion objective (0.3 NA, UMPLFLN, Olympus, Corp.) was used for GCaMP5G-expressing fly brain imaging. A 2× objective (0.14 NA, XLFluor2X/340, Olympus, Corp.) was used for GCaMP6f-expressing mouse brain slice imaging.

### 2.7 Photoacoustic Microscopy of GCaMP Mouse Brain during Hindpaw Stimulations

To further validate our technique for *in vivo* GCaMP imaging in mouse brains, we used the same PAM system as described for fruit fly imaging. Anesthesia was induced by ketamine (100  mg/mL) and xylazine (20  mg/mL) and maintained with 0.5% to 1% isoflurane in oxygen, with ventilator support and supplemental temperature support. The scalp of the mouse brain was cut open to expose the skull for photoacoustic imaging and was surgically sutured after the experiment. During the imaging, the mouse was secured using a tooth bar on a platform. Forepaw stimulations were induced by a needle electrode inserted under the skin of the left forepaw. The electrode was connected to a function generator (DS345, Stanford Research Systems) for generating stimulation pulses. All of the animal experimental procedures were in conformance with the laboratory animal protocol approved by Washington University in St. Louis.

We first acquired the vasculature map of the mouse brain expressing GCaMP6s and then monitored the PA response of the mouse brain at the selected point (marked by a white cross) during left hindpaw stimulation trials. Each stimulation trial consisted of 5 s of pulsed stimulation (300-μs pulse width and 1-mA amplitude at 3 Hz). The resting period after each stimulation period was 20 s. The PA signal change during stimulations was averaged over seven stimulation trials and then lowpass filtered with a cutoff frequency of 5 Hz.

### 2.8 Data Analysis

Image processing was performed in MATLAB (Mathworks, Inc.). In all experiments, the baseline PA and fluorescence signals were calculated as the averaged signals acquired before the simulation. The fractional changes in PA and fluorescence signals, compared with the baseline values, were then calculated.

## 3 Results

### 3.1 PAM of GCaMP5G-expressing Fruit Fly Brains *in Vivo*

One challenge for imaging GCaMP in live brains with PAT is the interference signals from hemoglobin, which has a strong optical absorption at the peak wavelength of the GCaMP absorption spectrum (488 nm). To overcome this challenge^64^ and unequivocally demonstrate the feasibility of GCaMP imaging with PAT, we chose a simple and well-established transgenic invertebrate model, *Drosophila* *melanogaster* (i.e., fruit fly), which does not have hemoglobin in its brain. For the proof-of-concept experiments, we used a transgenic fly line that expresses GCaMP5G pan-neutrally in all cholinergic neurons.

To image neural calcium signals in the fly brain, we developed a PA and epi-fluorescence dual-modality microscope [Fig. 1(a)]. The dual-modality microscope simultaneously recorded the odor-evoked neural activities in the fly brain *in vivo*, with an automatically coregistered field of view and complementary image contrasts. When a 488-nm–wavelength laser pulse excites the GCaMP molecules in the brain, the captured laser energy is transformed partially into fluorescence signals and partially into PA signals, with a 67% fluorescence quantum yield.^65^ It is worth noting that the ultrasonic transducer in our microscope temporally resolves PA signals from different depths along the acoustic axis, and no additional depth-scanning is required. Moreover, only a 2D raster scan is needed for PAM to form a 3D image. The PAM system and the epifluorescence microscopy (EFM) system share the same optical focusing pathway and thus have the same lateral resolution. The axial resolution of PAM is acoustically determined, whereas EFM lacks axial resolution over planar targets.

**Fig. 2:**
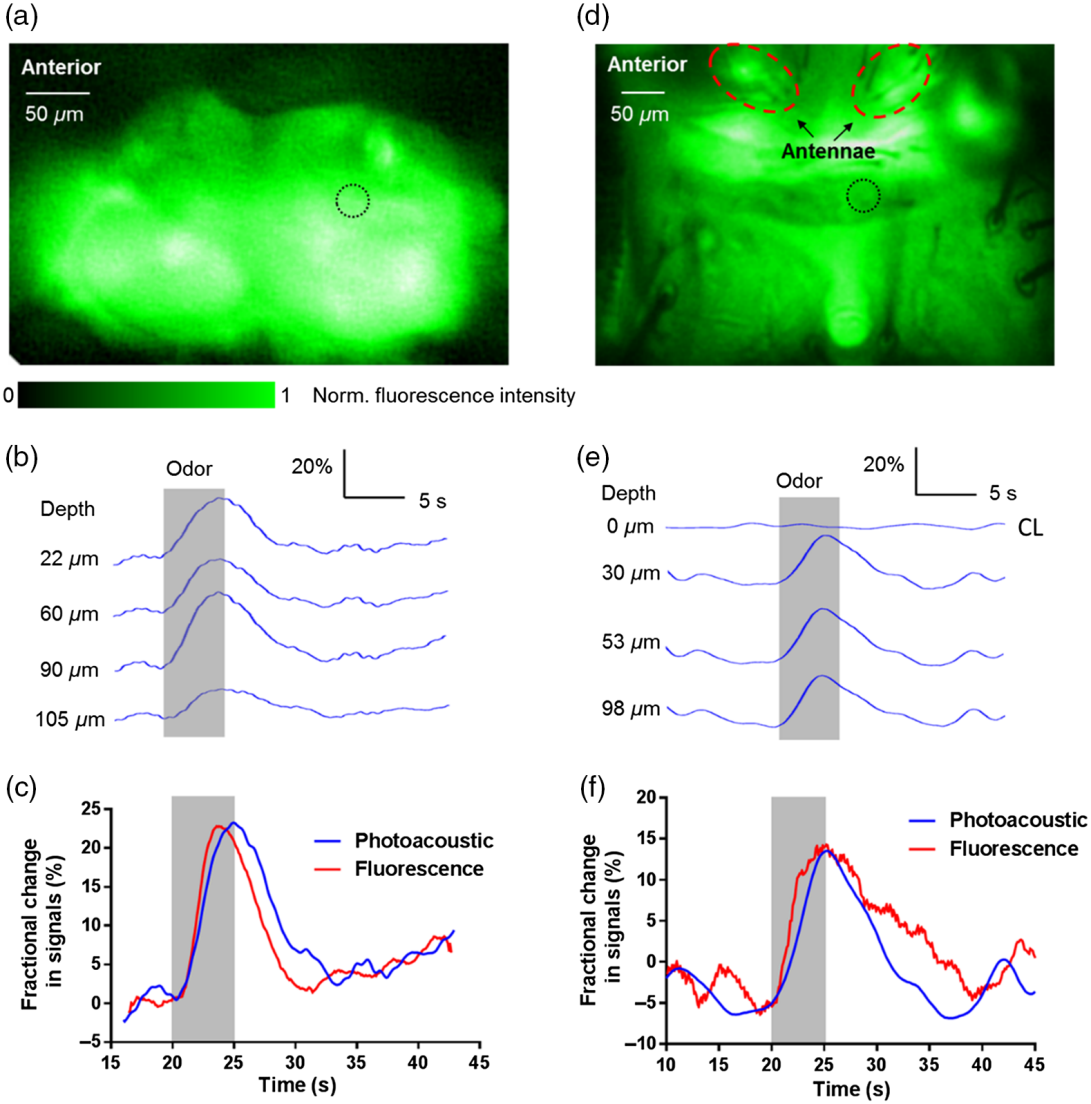
PA imaging of odor-evoked neural activity in the fly brain without and with cuticle. (a) Epi-fluorescence image of a GCaMP5G-expressing fly brain with the cuticle surgically removed. The dashed circle identifies the lateral location of M-mode PA recording. (b) Fractional PA amplitude changes from representative neuron layers in the glomerular region of the fly antennal lobe, showing varied response amplitudes and durations at different depths. Note that the depth in this panel is measured from the surface of the fly brain. The gray bar indicates when a puff of ethyl acetate was delivered to the fly antenna. (c) Averaged fractional PA amplitude response and the concurrently recorded fluorescence intensity response to odor stimulation with the cuticle removed. (d) Epifluorescence image of a GCaMP5G-expressing fly brain with the cuticle intact (i.e., not surgically removed). The lateral location of the M-mode PA recording is marked by the dashed circle. (e) Fractional PA amplitude changes from representative neuron layers in the fly brain with its cuticle intact. Note that the lack of response in the cuticle layer (CL) is shown here as a control. Depth in this panel is measured from the cuticle layer. The gray bar indicates the duration of the ethyl acetate delivery to the fly antennae. (f) Averaged fractional PA amplitude response from the fly brain with intact cuticle and the concurrently recorded fluorescence intensity response to odor stimulation. Although the fractional changes in PA amplitude and fluorescence intensity are comparable in magnitude, depth-resolved measurements of neural activity were made only by the PA system. (Video 1, MP4, 286 KB [URL: https://doi.org/10.1117/1.JBO.27.9.096004.s1]; Video 2, MP4, 640 KB [URL: https://doi.org/10.1117/1.JBO.27.9.096004.s2)

Using PAM, we first imaged the whole fly brain with the cuticle intact as well as the adjacent body parts. A maximum amplitude projection (MAP) image clearly shows the brain cuticle, antennae, compound eyes, and thorax [Fig. 1(b)]. The strong PA signals mostly come from the pigments of the cuticle. To gain direct optical access to neurons in the fly brain, we removed the cuticle posterior to the two antennae and the underlying connective tissue surrounding the brain.^59^ The PA depth-resolved images of the exposed fly brain show heterogeneous GCaMP expression levels in the brain [Fig. S1(a) in the Supplemental Material]. The strongest PA signals came from putative neural cell bodies. To confirm that the PA signal was generated by GCaMP, we used PAM to measure the absorption spectrum of the brain tissue by tuning the laser wavelength from 410 to 550 nm [Fig. S1(b) in the Supplemental Material]. The spectrum measured by PAM agrees well with that measured purely optically elsewhere.^65^ This result is strong evidence that GCaMP is the major chromophore in the fly brain in the visible spectral range. In addition, fluorescence signals from GCaMP were simultaneously acquired by the EFM channel [Fig. 2(a)] to further confirm the origin of the PA signals.

We monitored neural activities in the fly brain evoked by a sensory stimulus. We administered a 5-s long puff of ethyl acetate to the fruit fly antenna while monitoring the changes in the GCaMP signals in the glomerular regions of the antennal lobe (a region where olfactory sensory neuron axon terminals converge and synapse with the downstream neural populations). M-mode recording (i.e., repeated 1D depth-resolved PA imaging at the same lateral location) was performed in the fruit fly antennal lobe. Depth-resolved PA signals from different layers in the brain (∼200-μm thick) were acquired at a 1D imaging rate of 1 kHz [Fig. 2(b)]. The increase in GCaMP’s optical absorption coefficient led to the same fractional PA and fluorescent signal changes. In response to the odor stimulation, an ∼23% increase was observed in depth-averaged PA signals [Fig. 2(c)], which indicated elevated neural activities (Video 1). The strongest fractional PA signal change (∼34%) was observed at 90-μm beneath the brain surface. We observed different response durations at different depths from the PAM channel, which clearly indicates the heterogeneity in the dynamics of odor-evoked responses from sensory neuron axonal terminals. Ethyl acetate strongly activates different types of sensory neurons, particularly those housed in the large basiconic sensilla. The axonal terminals of these sensory neurons are known arborize in different glomeruli at different depths in the fly antennal lobe.^66^^,^^67^ The PA response time course averaged over all depths correlates well with the fluorescence response [Fig. 2(c)] simultaneously recorded at the same lateral location. This correlation indicates that the same neuronal events were being imaged by both the PAM and EFM channels. More importantly, depth-resolved signals were only obtained using PAM, clearly illustrating the benefit of the proposed approach. We also recorded similar PA and fluorescence responses in the lateral horn region of the fly brain (Fig. S2 in the Supplemental Material).

Next, taking advantage of the depth-resolving and scattering-tolerant capabilities of PAM, we sought to determine whether such depth-resolved signals can be obtained from flies with intact cuticles (i.e., without any surgical preparations) [Fig. 2(d)]. Although light was attenuated by the cuticle and connective tissue, PAM still detected signals from all neuron layers in the brain. We recorded concurrent M-mode PA and fluorescence signals upon odor stimulation [Fig. 2(e), Video 2). Similar to the results acquired with the cuticle removed, different PA responses were recorded from different neuronal layers [Fig. 2(e)]. The PA signals from the surface layer of the cuticle, serving as a built-in control, did not show any changes during odor stimulation, as expected. Note that, even with the intact cuticle, the fractional signal change was observed reliably [Figs. 2(e) and 2(f), Fig. S3 in the Supplemental Material]. Again, the PA results were confirmed by the concurrent fluorescence recording [Fig. 2(f)].

**Fig. 3:**
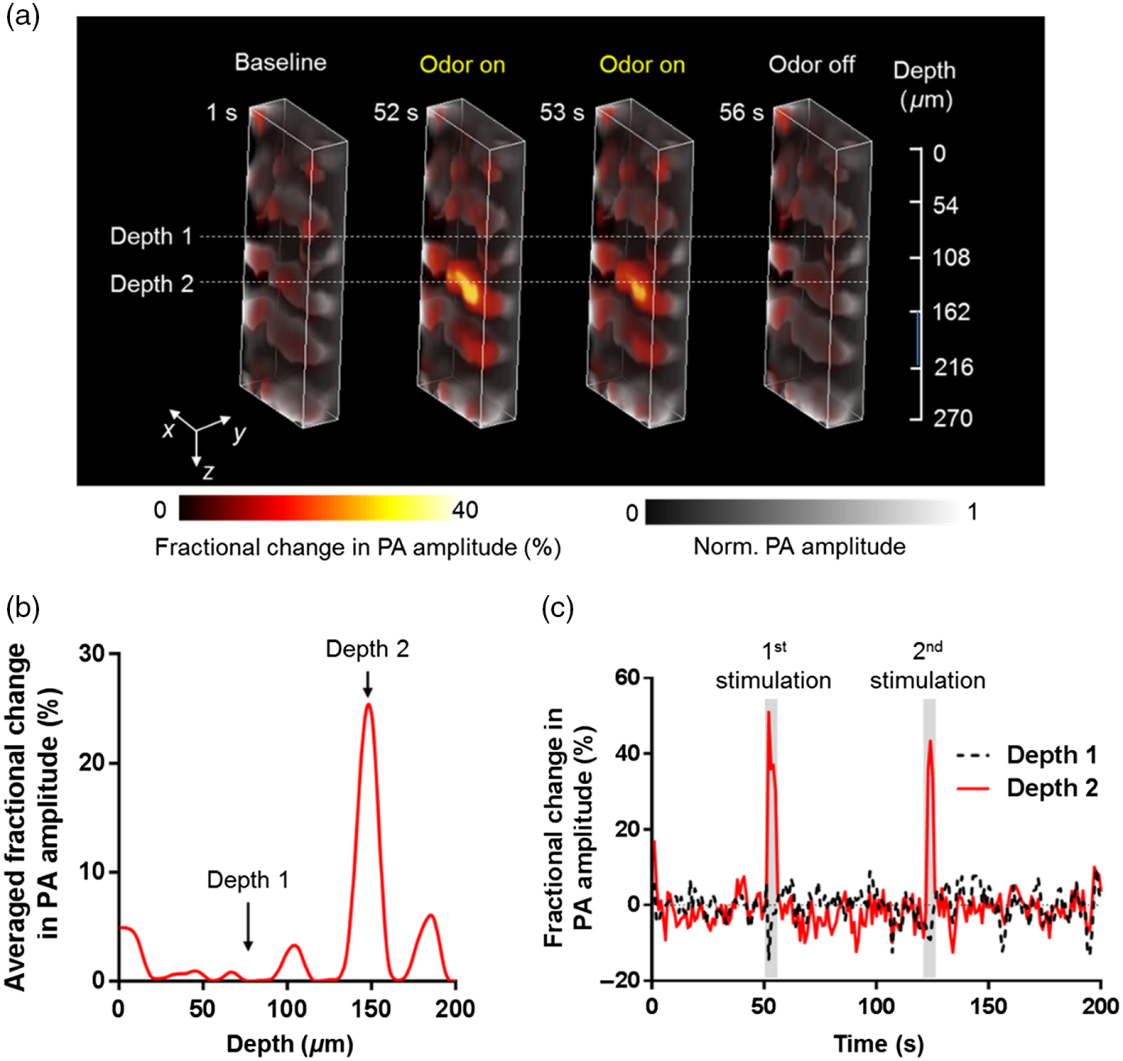
Volumetric PAM of odor-evoked neural activities in the glomerular antennal lobe region of a fly antennal lobe. (a) Volumetric images of the fly brain with the cuticle removed, before, during, and after two odor stimulations. The fractional changes in PA amplitude (shown in color) are overlaid on the structural image (shown in gray). (b) Depth-wise averaged fractional changes in PA amplitude during odor stimulations. The changes are averaged over the two stimulations. (c) Time courses of averaged fractional PA amplitude changes from two representative layers [labeled in (a)] of neurons. The gray bars indicate two successive puffs of ethyl acetate. (Video 3, MP4, 502 KB [URL: https://doi.org/10.1117/1.JBO.27.9.096004.s3]; Video 4, MP4, 392 KB [URL: https://doi.org/10.1117/1.JBO.27.9.096004.s4])

As mentioned earlier, PAM can render a 3D calcium image with only a 2D scan. To illustrate this, we imaged a volume of 100  μm (x axis) by 40  μm (y axis) by 270  μm (z axis) in the antennal lobe region of the fly brain (with the cuticle removed), at a volumetric imaging rate of 1 Hz (Fig. 3, Fig. S4 in the Supplemental Material, and Videos 3 and 4). Both anatomical structures and the functional activity of neurons expressing GCaMP5G in the putative glomerular layers of the fly antennal lobe were clearly imaged [Fig. 3(a)]. The diversity of odor-evoked responses at different depths was still captured [Figs. 3(b) and 3(c)]. In sum, these results convincingly show that PAM can monitor depth-resolved calcium signals from neurons with high spatial and temporal resolutions.

### 3.2 PACT of Live GCaMP6f-Expressing Mouse Brain Slices *ex Vivo*

**Fig. 4:**
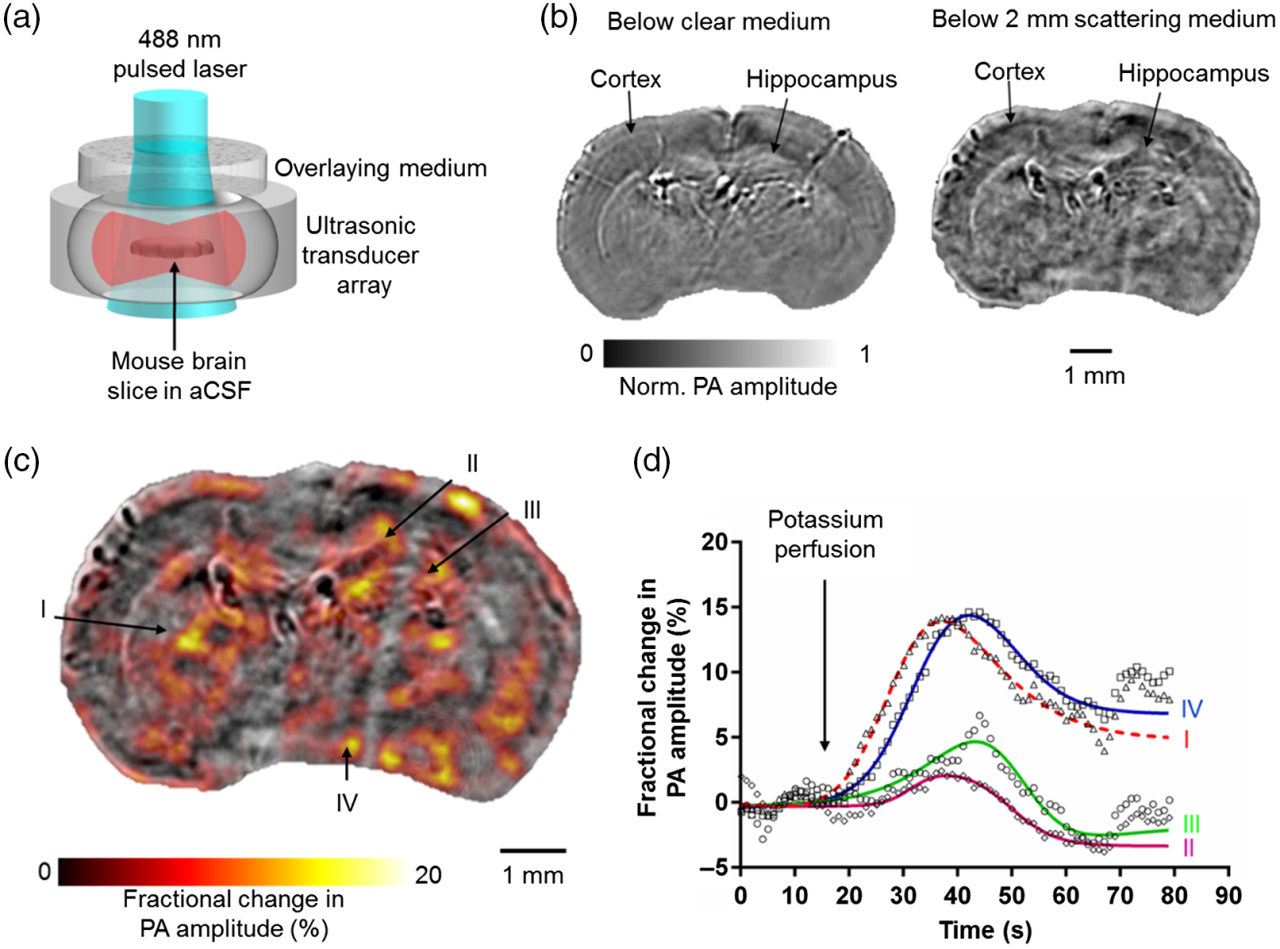
PACT monitoring of *in vitro* neural activities in GCaMP6f-expressing mouse brain slices. (a) Schematic of the high-speed PACT system, with wide-field illumination and parallel acoustic detection. The GCaMP6f-expressing brain slice was immersed in a regular aCSF solution in the imaging chamber. (b) PACT images of the GCaMP6f-expressing brain slice in a clear medium (left) and through a 2-mm-thick scattering Intralipid solution (right, reduced optical scattering coefficient $10\;{\mathrm{cm}}^{-1}$). (c) PACT of the neural activity in a GCaMP6f-expressing mouse brain slice in response to high-potassium perfusion. The fractional PA amplitude change (shown in color) is superimposed on the baseline image (shown in gray). (d) Time courses of fractional PA amplitude changes from four representative regions (I, II, III, and IV) as indicated in (c). The solid lines are fittings using a bell-shaped dose-response model. (Video 5, MP4, 2046 KB [URL: https://doi.org/10.1117/1.JBO.27.9.096004.s5]; Video 6, MP4, 265 KB [URL: https://doi.org/10.1117/1.JBO.27.9.096004.s6])

Taking advantage of PACT’s deep imaging capability, we used our recently developed high-speed PACT system to image neural activity at depths greater than the optical diffusion limit of ∼1  mm [Fig. 4(a)].^68^ With wide-field illumination and parallel acoustic detection, the newly developed PACT system features high spatiotemporal resolution, deep penetration, and full-view fidelity. PACT acquires a 2D cross-sectional image with a 100-μm resolution at a 10-Hz frame rate. Further, only a single laser pulse is used to generate each 2D image, thereby greatly reducing unnecessary light exposure and resultant photobleaching. In the absence of hemodynamically induced PA signal changes in the brain slice, GCaMP6f is the chromophore that can generate dynamic PA signals upon 488-nm laser excitation during neural activities.

We imaged neural activity in acute coronal slices (300-μm thick) of a GCaMP6f-expressing mouse brain. The brain slice was mounted in the PACT imaging chamber, which was filled with regular aCSF, and imaged either without any scattering medium or through a 2-mm-thick tissue-simulating scattering medium [Fig. 4(b)]. In PACT images acquired without an overlying scattering medium, major brain structures expressing GCaMP6f, such as the cortex and hippocampus, were clearly resolved. Notably, even when the brain slice was covered with a 2-mm-thick scattering medium (1% Intralipid in deionized water, with a reduced scattering coefficient $∼10\;{\mathrm{cm}}^{-1}$), GCaMP6f-rich brain structures were still observable [Fig. 4(b)].

To stimulate the neurons in the brain slice, we performed high-concentration potassium perfusion in the PACT imaging chamber. The elevated extracellular potassium depolarized neurons in the brain slice and induced action potentials. Upon high-potassium perfusion, increased PA signals were recorded from multiple areas within the brain slice [Fig. 4(c), Video 5]. The fractional PA amplitude changes shown in Fig. 4(d) were averaged over representative regions of interest. The fractional PA amplitude increased by up to ∼15% during the potassium perfusion. After the potassium perfusion, the intracellular calcium concentration gradually returned to the baseline level through diffusion and sodium-calcium exchanges, resulting in decreased PA amplitudes in the originally responding areas. In another experiment with a mouse brain slice covered with 1.5-mm-thick mouse brain tissue, we also observed a similar PA signal increase upon high-potassium perfusion. The PA response was independently validated by monitoring the fluorescence response of a brain slice in regular aCSF, measured with a commercial wide-field fluorescence microscope. The same sample preparation and stimulation procedures were used (Fig. S5 in the Supplemental Material and Video 6). In sum, our results confirm that PACT is a unique and effective calcium imaging technique with an image depth beyond the optical diffusion limit.

### 3.3 PAM of a GCaMP6s-Expressing Mouse Brain *in Vivo*

In addition to optical scattering, another obstacle to photoacoustic neural imaging in mammalian brains is the presence of strong hemodynamic signals, which may overshadow the changes in GCaMP signals. By imaging spatially confined areas with minimal vasculature, the contribution of hemodynamics can be minimized. In our pilot study, we first acquired the vasculature map of a mouse brain expressing GCaMP6s [Fig. S6(a) in the Supplemental Material]. Then we monitored the PA signal change of a selected point with minimal vasculature over the stimulation trials. It can be seen that the local PA response from the mouse brain *in vivo* shows a relatively slow increase over the 5-s stimulation period [Fig. S6(b) in the Supplemental Material] with 15 stimulation pulses at 3 Hz. Interestingly, after the start of each stimulation pulse, a fast increase in PA signal can be observed on top of the slow signal change. Considering the relatively fast temporal response of GCaMP compared with that of hemodynamic signals, we believe that the fast increase in PA signals came from GCaMP and the slow change came from the hemodynamic change.

## 4 Discussion

We have demonstrated, for the first time, that multiscale PAT can be used for imaging neuronal calcium signals, with high spatiotemporal resolution and deep penetration. With fast dynamics, high photostability, and a large fractional absorption response, GCaMP or its variants offers an ideal optical contrast for PAT as the PA signal is exquisitely sensitive to optical absorption. With the depth-resolved imaging capability, we showed that PAM can perform fast 3D calcium imaging in fruit flies expressing genetically encoded calcium indicators. Notably, using PAM, we were able to monitor depth-resolved odor-evoked neural activities from the fly brain, without the need for invasive surgery, which could compromise experimental reproducibility.^69^ For *in vivo* mouse brain imaging, we showed that GCaMP signals can be temporally separated from hemodynamic signals. To unambiguously differentiate hemodynamic signals and GCaMP signals *in vivo*, multiwavelength PAT can be implemented to simultaneously record hemodynamic and GCaMP signals, enabling studies of neurovascular coupling. Although we focused on the feasibility of PAM for neural imaging here, the imaging rate can be readily increased by utilizing a higher pulse-repetition-rate laser and an optimized scanning mechanism.^70^^–^^72^

Optical scattering of light severely limits high-resolution optical imaging techniques to superficial tissue layers. Our results clearly demonstrate that, by utilizing diffused photons, PACT can image neural responses beyond the optical diffusion limit. Prior work has shown that PACT can provide a penetration depth of 8 mm in a rat brain.^68^ Although, the imaging depth of PACT demonstrated in this study was largely limited by the relatively short absorption wavelength of GCaMP, it can be further improved using calcium indicators that absorb strongly at longer wavelengths,^52^^,^^73^^,^^74^ especially in the near-infrared wavelength region (700 to 1100 nm). The combination of near-infrared calcium indicators and PACT can potentially enable small-animal whole-brain neural imaging.

So far, a variety of imaging modalities have been explored for brain studies, especially two-photon microscopy and functional magnetic resonance imaging (fMRI). At the microscopic scale, two-photon microscopy allows for detailed and direct examination of neural activities in small animal models.^75^ At the macroscopic scale, fMRI has been extensively used to map human or large-animal brain activities, allowing for indirect coarse-grained localization of cognitive functions with hemodynamic signals. However, correlating the two imaging scales still remains a major challenge due to the different signal-contrast sources of these two modalities. Therefore, there is a gap in our understanding between the macroscopic activity patterns in humans or large animals and the microscopic activity details in small-animal models. PAT has been scaled up for multiple-centimeter penetration and can provide multiscale imaging for not only small animals but also potentially large animals (at least with open skulls).^76^^,^^77^ Multiwavelength PAT can be implemented to simultaneously record and unmix hemodynamic and calcium signals, enabling studies of neurovascular coupling. Therefore, PAT is uniquely positioned to bridge this contrast gap across the length scales, facilitating correlative neuroscience studies in various animal models of brain diseases such as Alzheimer’s disease and stroke.^7^

We have shown that PAT holds great promise for neuronal calcium imaging in the brains of transgenic invertebrate and vertebrate models expressing GCaMP indicators. With PAM, we demonstrate *in vivo* high-resolution depth-resolved neural imaging without depth scanning that enables 3D imaging of neural activities at high temporal resolution. With PACT, we demonstrate neural activity imaging with GCaMP beyond the optical diffusion limit. The combination of advanced PAT technologies and genetically encoded neural activity indicators optimized for PA imaging offers the neuroscience community a methodology for studying neural networks *in vivo*.

## 5 Conclusion

We have shown that PAT holds great promise for neuronal calcium imaging in the brains of transgenic invertebrate and vertebrate models expressing GCaMP indicators. With PAM, we demonstrate *in vivo* high-resolution depth-resolved neural imaging without depth scanning that enables 3D imaging of neural activities at high temporal resolution. With PACT, we demonstrate neural activity imaging with GCaMP beyond the optical diffusion limit. The combination of advanced PAT technologies and genetically encoded neural activity indicators optimized for PA imaging offers the neuroscience community a methodology for studying neural networks *in vivo*.
