# Supplementary material for: Multiscale photoacoustic tomography of neural activities with GCaMP calcium indicators
Source: J Biomed Opt. 2022 Sep 10;27(9):096004. doi: 10.1117/1.JBO.27.9.096004 (PMC9463545; doi:10.1117/1.JBO.27.9.096004)
Supplement: Supplementary file 1 [file JBO_027_096004_SD001.pdf]

SUPPLEMENTARY FIGURES AND CAPTIONS

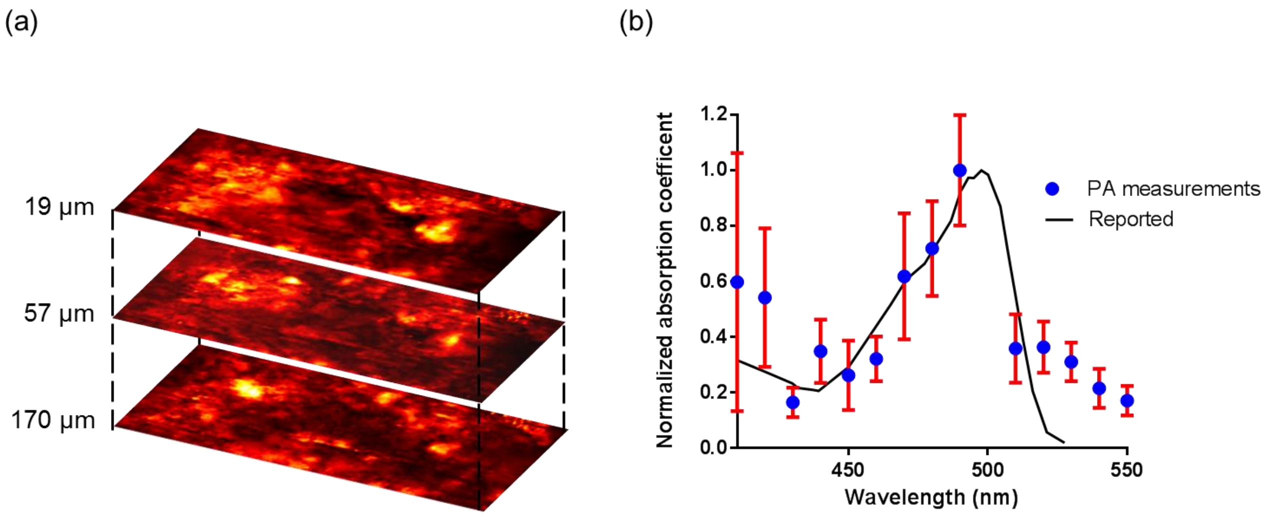

Supplementary Fig. 1. Spectral characterization of a fly brain. (a) Cross-sectional photoacoustic microscopy images of the brain at different depths, acquired at 488 nm. The cuticle was surgically removed. The labeled depths indicate the axial distances from the brain surface. (b) PA spectrum (blue dots) of the fly brain, which correlates well with the reported absorption spectrum (black curve) of GCaMP5G[43].

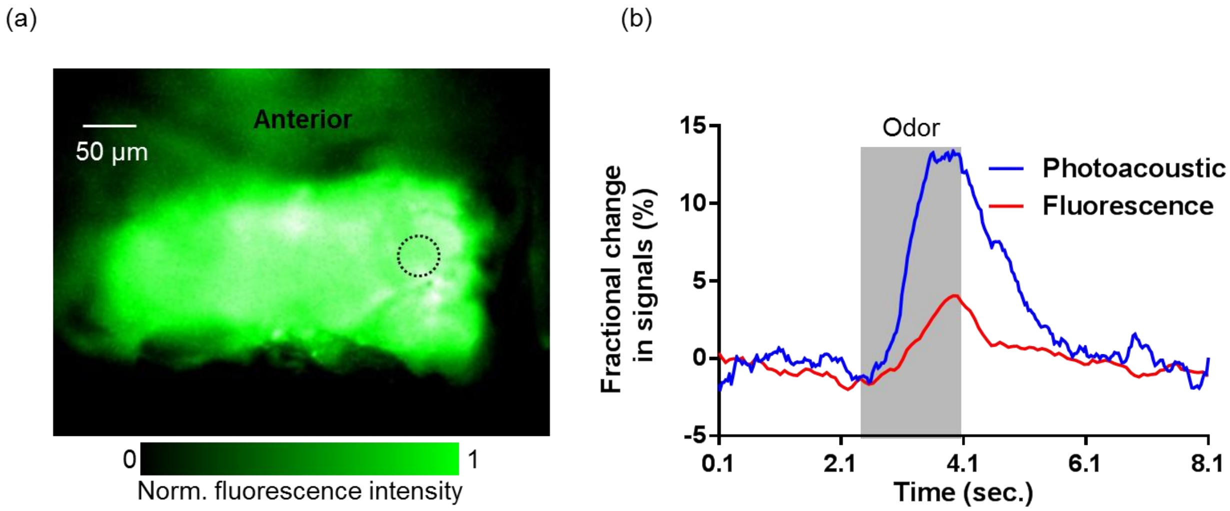

Supplementary Fig. 2. Epi-fluorescence imaging and M-mode PA recording of odor-evoked neural activities in the lateral horn region of a fly brain. (a) Epi-fluorescence image of a GCaMP5G-expressing fly brain with cuticle removed. The location of the M-mode PA recording is marked by the dashed circle. (b) Averaged fractional PA signal change and concurrent fluorescence response to odor stimulations in the fly brain, with cuticle removed.

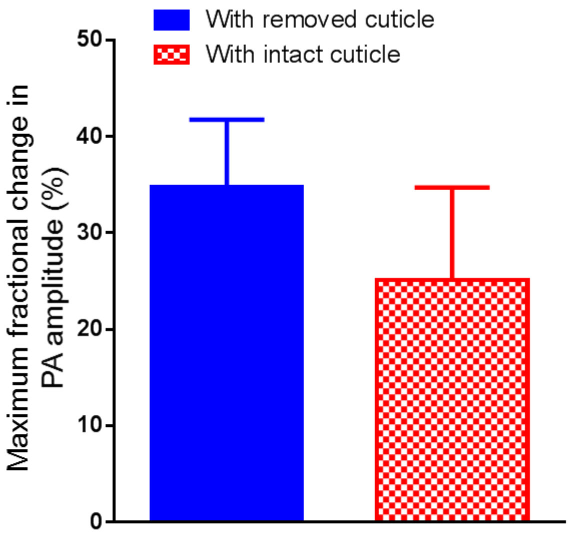

Supplementary Fig. 3. Odor-evoked maximum fractional change in PA amplitude in the fly brain, with removed and intact cuticle, respectively. Error bars, standard deviations. n = 3.

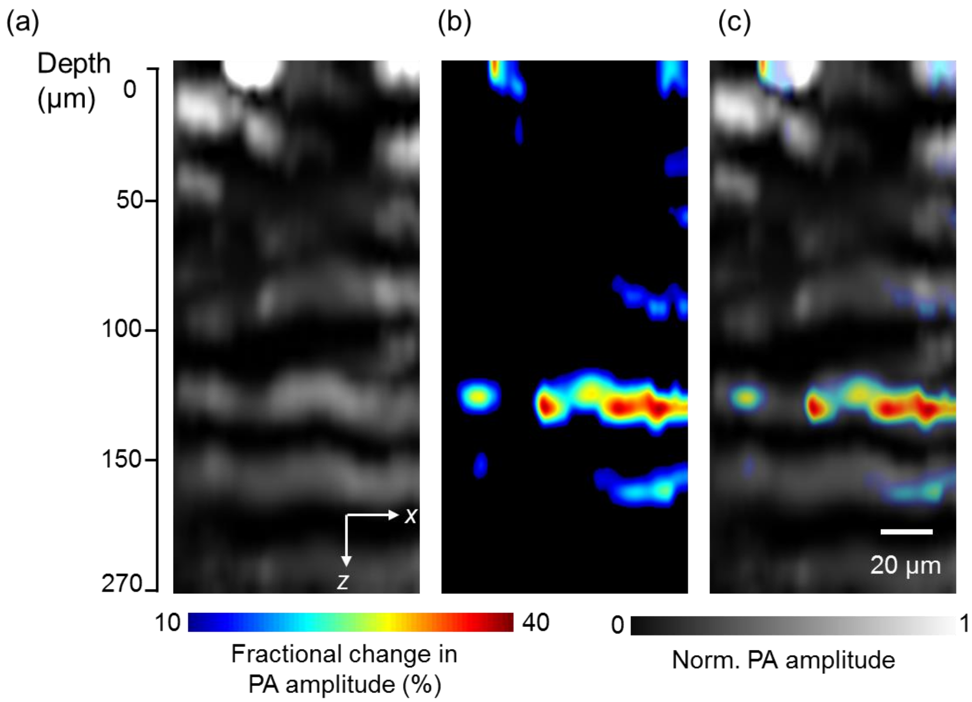

Supplementary Fig. 4. PAM of odor-evoked neural activity in the antennal lobe region of a fly brain with the cuticle removed, in a cross-sectional view. (a) Cross-sectional structural image of the antennal lobe region of a fly brain with the cuticle removed. (b) Fractional change in PA amplitude in the region shown in (a), during odor stimulation. (c) Fractional change in PA amplitude overlaid with the cross-sectional structural image of the fly brain.

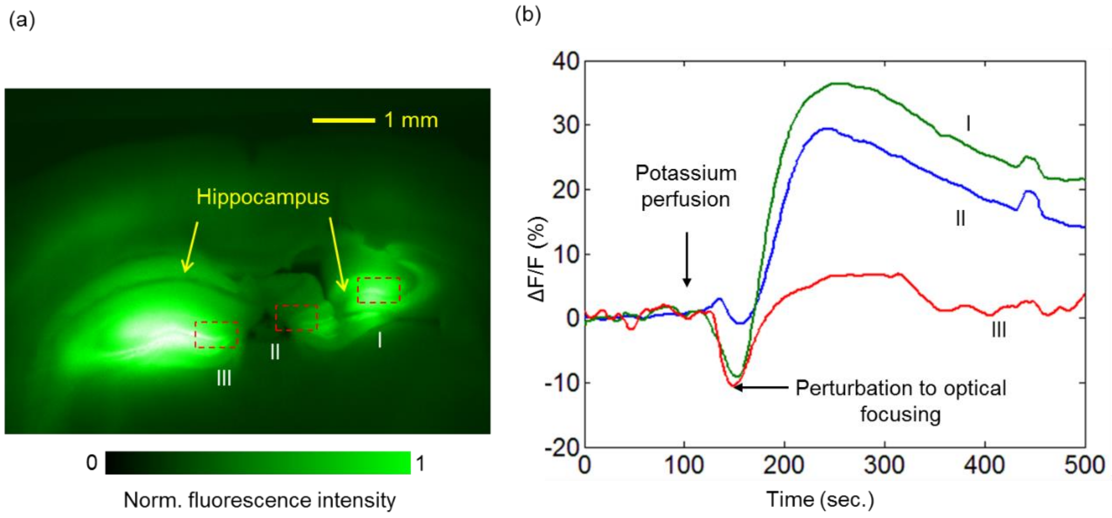

Supplementary Fig. 5. Wide-field fluorescence microscopy of a GCaMP6f-expressing mouse brain slice. (a) Fluorescence image of a GCaMP6f-expressing mouse brain slice, showing that the hippocampus area had a high expression level of GCaMP6f. (b) Averaged fractional fluorescence intensity changes after high-potassium perfusion, from the three regions marked in (a). The short-period signal decrease right after the perfusion started was due to optical defocusing caused by the perfusion perturbation.

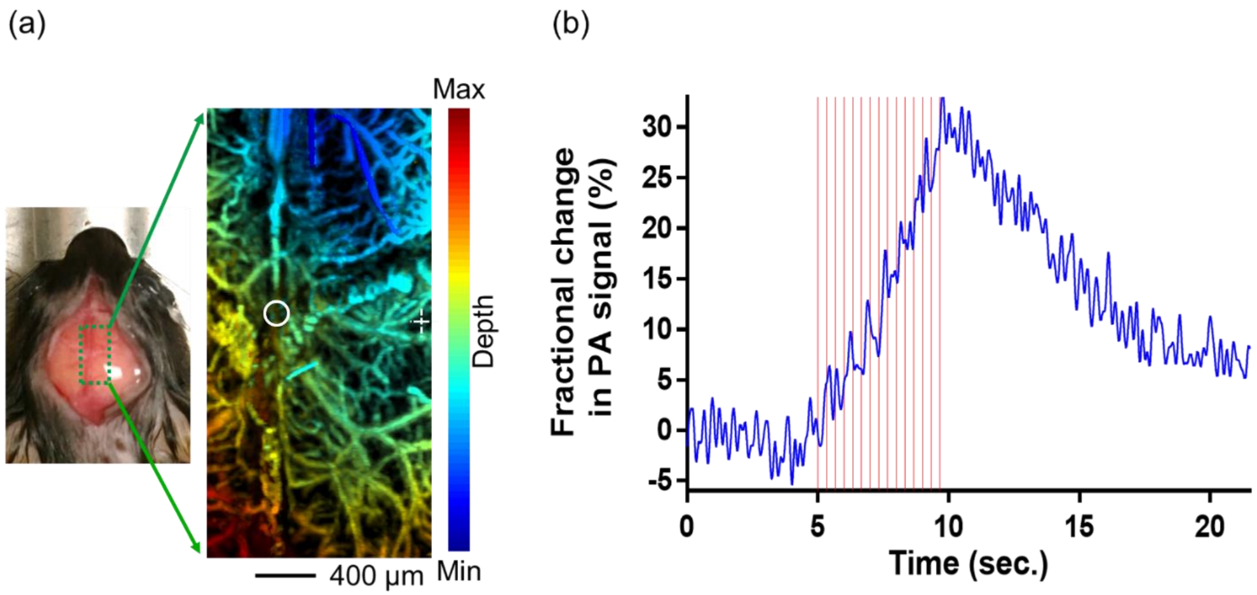

Supplementary Fig. 6. *In vivo* photoacoustic microscopy of a GCaMP6f-expressing mouse brain during left hindpaw stimulation. (a) Photoacoustic microscopy of the vasculature of the GCaMP mouse brain acquired at 532 nm. Bregma is labeled by the white circle. (b) PA amplitude change at 488 nm averaged over 7 consecutive paw stimulation trials. Each vertical red line corresponds to the start of a stimulation pulse with 300-μs pulse width and 1-mA amplitude.

SUPPLEMENTARY VIDEOS

Supplementary video 1. PA (yellow curve) and fluorescence responses (shown in a multi-color image and a green curve) to odor stimulations in the antennal lobe region in a fly brain with the cuticle removed.

Supplementary video 2. PA (yellow curve) and fluorescence responses (shown in a multi-color image and a green curve) to odor stimulations in the antennal lobe region in a fly brain with the cuticle intact.

Supplementary video 3. Volumetric PAM of odor-evoked neural activity (shown in color) in a fly brain with the cuticle removed.

Supplementary video 4. PAM of odor-evoked neural activity in a fly brain in the cross-sectional view, with the cuticle removed.

Supplementary video 5. PACT of high-potassium perfusion induced neural activity in a live mouse brain slice, through a 2-mm-thick tissue-simulating scattering medium.

Supplementary video 6. Fluorescence microscopy of high-potassium perfusion induced neural activity in a live mouse brain slice.
